# A One Health Approach to Hepatitis E Virus in Venezuela: Low Seroprevalence in Humans and First Genomic Evidence of Hepatitis E Virus Genotype 3 in a Domestic Swine

**DOI:** 10.3390/microorganisms14051045

**Published:** 2026-05-06

**Authors:** Julie Andreina Beltrán, Yoneira Fabiola Sulbarán, Lily Soto, Carlos Pérez, Mario Comegna, María Graciela López, Nahir Martínez-Urbina, Moraima Hernández, Marjorie Bastardo-Méndez, Alejandra Zamora-Figueroa, Mariana Hidalgo, Flor Helene Pujol, Rossana Celeste Jaspe

**Affiliations:** 1 Laboratorio de Virología Molecular, Centro de Microbiología y Biología Celular (CMBC), Instituto Venezolano de Investigaciones Científicas (IVIC), Caracas 1020-A, Venezuela; beltranjulie@gmail.com (J.A.B.); yfsulbara@gmail.com (Y.F.S.); alejandra.zamora@gmail.com (A.Z.-F.); 2 Unidad de Microscopía Electrónica, Centro de Química, Instituto Venezolano de Investigaciones Científicas (IVIC), Caracas 1020-A, Venezuela; 3 Sección de Inmunología, Instituto de Medicina Tropical, Universidad Central de Venezuela (UCV), Caracas 1050, Venezuela; dralilysoto@gmail.com; 4 Unidad de Infectología, Hospital Dr. Jesús Yerena, Lídice, Caracas 1010, Venezuela; cpperez2000@yahoo.com; 5 Fundación Proyecto Once Trece, Caracas 1060, Venezuela; mcomegna20076@gmail.com; 6 Unidad de VIH, Servicio de Enfermedades Infecciosas, Hospital de Niños J. M. de los Ríos, Caracas 1011, Venezuela; magrelopez@gmail.com; 7 Sección de Virología, Instituto de Medicina Tropical, Universidad Central de Venezuela (UCV), Caracas 1050, Venezuela; nahir.martinez@ucv.ve; 8 Unidad de Infectología, Maternidad Concepción Palacios, Caracas 1010, Venezuela; moraima_h@yahoo.es; 9 Laboratorio de Ecología de Microorganismos, Centro de Ecología Aplicada, Instituto de Zoología y Ecología Tropical, Facultad de Ciencias, Universidad Central de Venezuela (UCV), Caracas 1050, Venezuela; marjoriedc66@gmail.com; 10 Laboratorio de Inmunoparasitología, Centro de Microbiología y Biología Celular (CMBC), Instituto Venezolano de Investigaciones Científicas (IVIC), Caracas 1020-A, Venezuela; mariana.hidalgo.r@gmail.com

**Keywords:** hepatitis E virus, seroprevalence, animal hosts, wastewater, complete HEV genome, genotype 3, genomic surveillance

## Abstract

Hepatitis E virus (HEV) is an emerging zoonotic pathogen of increasing concern in developed regions and represents a major cause of acute viral hepatitis worldwide, primarily transmitted via the fecal–oral route. Although most infections are self-limiting, immunocompromised individuals, such as people living with human immunodeficiency virus (PLWH) and pregnant women, are at risk of severe outcomes, including chronic infection and fatal liver failure, respectively. This study was aimed at evaluating the prevalence and genetic diversity of HEV in PLWH and relevant ecological niches (swine and wastewater) in Venezuela. A total of 417 serum samples from PLWH, 85 wastewater samples, and 67 swine fecal samples were tested for serological or molecular HEV markers. The seroprevalence of anti-HEV antibodies among PLWH was 0.2% for IgM and 5.5% for IgG. HEV RNA was not detected in samples from PLWH or wastewater; however, a 1.5% prevalence of active infection was identified in swine. Phylogenetic analysis of a complete HEV genome revealed an unassignable subtype within genotype 3, tentatively designated as 3p. To the best of our knowledge, this study provides the first molecular characterization and report on HEV frequency in PLWH, wastewater, and swine in Venezuela.

## 1. Introduction

Hepatitis E virus (HEV) is a quasi-enveloped RNA virus belonging to the *Hepeviridae* family, *Orthohepevirinae* subfamily, and *Paslahepevirus balayani* species. HEV infection is a leading cause of acute viral hepatitis worldwide, accounting for an estimated 20 million annual infections [1]. This infection is recognized as an emerging zoonotic disease of increasing concern in developed countries. In highly endemic regions, transmission occurs primarily via the fecal–oral route, often through contaminated water or food. Conversely, in industrialized countries, infection is mainly associated with the consumption of raw or undercooked meat from infected animals [2].

HEV infects humans and a broad range of mammalian hosts, exhibiting a heterogeneous worldwide distribution [3]. Its genome is approximately 7.2 kb long and contains three primary open reading frames (ORFs), with a fourth ORF unique to genotype 1 [4]. ORF1 encodes a nonstructural polyprotein, ORF2 encodes the capsid protein, and ORF3 encodes a multifunctional protein [5]. To date, up to eight genotypes (HEV-1 to HEV-8) have been described [3,6]. Genotypes 1 and 2 are obligate human pathogens and are the most common causes of epidemic and endemic hepatitis E in developing regions [7]. In contrast, genotypes 3, 4, and 7 are zoonotic and are transmitted via undercooked meat, animal contact, or contaminated blood products, with domestic pigs and wild boars serving as the primary reservoirs [8]. Genotypes 5 and 6 infect wild boars, whereas genotypes 7 and 8 have been identified in dromedaries [9]. HEV-3, which has a worldwide distribution, is considered to have the highest genetic diversity, comprising 16 recognized subtypes (3a-3o, 3ra) and multiple lineages [2,3,10]. Recent studies in Latin America have proposed novel HEV-3 subtypes, highlighting the region’s distinct viral ecology and the need for enhanced local genomic surveillance [11].

In immunocompetent individuals, HEV typically causes an acute, spontaneously resolving infection that lasts for approximately 2–9 weeks [12]. In contrast, immunocompromised patients, such as solid organ transplant recipients, individuals with hematologic malignancies undergoing chemotherapy, and people living with HIV (PLWH), are at significant risk of progressing to chronic HEV infection related to genotypes 3, 4, and 7. Chronic infection can lead to progressive liver fibrosis and cirrhosis, which can be fatal [2,13,14,15,16,17]. Severe outcomes, including fulminant hepatic failure, are strongly associated with infection during pregnancy, particularly with genotypes 1 and 2, where maternal mortality rates can reach 30% in the third trimester [2,18,19,20]. Premature birth, low birth weight, and fetal death have also been documented [19]. Furthermore, numerous extrahepatic manifestations have been described in patients with HEV infection [21,22].

In Latin America, HEV is widely distributed; however, its overall epidemiology remains poorly characterized, and the burden of the disease remains largely unknown. In South America, the virus has been detected in humans, swine, wild boars, environmental samples (water and sewage), and food [23]. Reported seroprevalence varies considerably by region and population studied, ranging from 0.1% to 38%. In blood donors, the seroprevalence ranges from 1.8% to 9.8%, whereas higher rates have been reported in PLWH, transplant recipients, and patients on hemodialysis [24]. Serological and molecular studies have confirmed that HEV-3 is the most frequently detected genotype in this region, followed by HEV-1 [23,24,25,26,27,28]. A high prevalence in swine has been widely reported, indicating extensive viral circulation within this animal reservoir [11,25,29].

Wastewater-based epidemiology (WBE) represents a noninvasive tool for monitoring community-level viral circulation. Environmental surveillance has offered insights into circulating viral subtypes and transmission links among humans, animals, and environmental interfaces [11,29,30]. A limited number of studies have reported HEV prevalence in environmental samples from Latin America [11,30,31,32,33,34]. This approach has not been systematically applied in Venezuela and could be pivotal for assessing real-time viral activity and genotypes, effectively complementing clinical surveillance.

Historically, Venezuela has been classified as a country with a low endemicity for HEV [35]. Available data remain limited, and HEV is considered a neglected pathogen. One study demonstrated that HEV is a relevant cause of acute liver disease in the country, and the detection of genotype 3 suggests a link to zoonotic reservoirs [36]. Implementing integrated epidemiological surveillance across human populations (especially PLWH), animal reservoirs (swine), and environmental matrices (wastewater) is paramount. In the context of Venezuela, a One Health Surveillance approach is essential.

This study was aimed at assessing the frequency of serological and molecular HEV markers in PLWH in the Capital District of Venezuela, complemented by parallel surveillance of swine and wastewater. Considering these aspects, this study provides the first serological evidence of HEV infection in PLWH, the first complete genome of HEV G3 from a domestic swine, and the first report of the virus in wastewater in Venezuela, representing a foundational application of the One Health approach.

## 2. Materials and Methods

This study was approved by the Human and Animal Bioethical Committees of the Venezuelan Institute for Scientific Research (IVIC) under ethical approval codes CD-2024/0874 and COBIANIM2025-04, respectively.

### 2.1. Blood Samples

A total of 417 blood samples were obtained from PLWH in Venezuela (291 males [70%] and 126 females [30%]; median age 36.6 ± 13.6 years) between 2022 and 2025 following written informed consent. The samples were stored at −80 °C until further analysis. This group included 33 children and adolescents (aged 10 months to 17 years) under care at the Infectious Diseases Unit of the J.M. de Los Ríos Children’s Hospital, and 18 pregnant women receiving obstetric care at the Infectious Diseases Service of the Concepción Palacios Maternity Hospital. Most participants were from the Capital District. For comparative purposes, an additional set of 18 serum samples was obtained from individuals without HIV residing in Bolívar State. This group comprised 6 pregnant women and 12 individuals who reported close contact with swine (Table 1).

### 2.2. Wastewater Samples

Eighty-five wastewater samples were processed, comprising 20 from 2022, 20 from 2023, 18 from 2024, and 27 from 2025. Samples collected between 2022 and 2023 were obtained from domestic effluent discharge points across several urban sectors of the Caracas metropolitan area as part of the project “Detection of SARS-CoV-2 in wastewater of Caracas.” The sampling frequency and physicochemical analyses were performed as previously described by Bastardo-Méndez et al. [37]. Samples from 2024 and 2025 were collected monthly directly from the Guaire River under the project “Environmental surveillance of poliovirus and other pathogens in wastewater.” Notably, the Guaire River receives untreated sewage due to a lack of operational wastewater treatment plants in the city. Physicochemical parameters were not measured for the 2024–2025 samples.

For all sampling events, a manual collection device was used to systematically retrieve samples from fixed points. Approximately 1 L of wastewater was collected, packed in sterile glass bottles, kept at 4 °C during transport, and processed upon arrival at the laboratory. Pasteurization and viral concentration were performed according to the protocols described by Bastardo-Méndez et al. [37].

### 2.3. Swine Fecal Samples

During 2024 and 2025, sixty-seven individual or pooled swine fecal samples were collected from the pen floor in one slaughterhouse and four family farms across several Venezuelan states: Miranda (*n* = 31), Aragua (*n* = 23), Guárico (*n* = 10), and Bolívar (*n* = 3). Samples were collected in sterile containers and immediately sent to the laboratory for processing. When immediate processing was not possible, the samples were frozen at –20 °C until analysis.

Fecal matter (0.5 g) was resuspended in 500 µL of sterile phosphate-buffered saline (PBS), vortexed vigorously for 10 min, and centrifuged at 14,000 rpm for 5 min. The resulting supernatant was stored at –80 °C until further analysis.

### 2.4. Serologic Markers

Serum samples were tested for anti-HEV IgM and IgG antibodies using a commercial enzyme-linked immunosorbent assay (ELISA) (EIAgen HEV kit, Adaltis, Milano, Italy), according to the manufacturer’s instructions. The cut-off was calculated as the mean optical density (OD) of the negative control (NC) plus 0.250 (IgM) or 0.350 (IgG). For IgM, the cut-off ranged from 0.392 to 0.410 (NC mean: 0.042–0.060; positive control (PC): 2.527–2.844); for IgG, it ranged from 0.301 to 0.320 (NC mean: 0.0505–0.0685; PC: 2.050–2.468). Results were interpreted using the sample-to-cut-off ratio (S/Co = sample OD/cut-off). For IgM, samples were considered negative if S/Co < 1.0, equivocal if 1.0–1.2, and positive if >1.2. For IgG, samples were considered negative if S/Co < 0.9, equivocal if 0.9–1.1, and positive if >1.1. Equivocal samples were re-tested. IgM-positive samples were confirmed with a second ELISA (RecombiLISA, CTK Biotech, Inc., Poway, CA, USA). Absorbance was determined using a microplate spectrophotometer SpectraMax250 ELISA reader (Molecular Devices, Sunnyvale, CA, USA) at a wavelength of 450 nm with a secondary filter of 620–630 nm.

The manufacturer of the ELISA kits reported high diagnostic accuracy for these assays. Specifically, the IgM assays demonstrated a sensitivity of 98% for the EIAgen kit and 100% for the RecombiLISA kit, while both achieved a specificity of 100%. Furthermore, the EIAgen HEV IgG assay exhibited an analytical sensitivity of approximately 0.1 WHO IU/mL, with a reported specificity of 100%.

### 2.5. Molecular Detection and Sequencing

Total RNA was extracted from all seropositive serum samples (one anti-HEV IgM-positive and 23 anti-HEV IgG-positive) and all swine fecal samples using the QIAamp Viral RNA Mini Kit (QIAGEN GmbH, Hilden, Germany), following the manufacturer’s instructions. Total RNA from wastewater samples was extracted using the Quick-DNA/RNA™ Viral Kit (Zymo Research Corp., Irvine, CA, USA) according to the manufacturer’s instructions. To mitigate PCR inhibition inherent to this complex matrix, RNA extracts from wastewater samples underwent an additional purification step using the OneStep™ PCR Inhibitor Removal Kit (Zymo Research Corp., Irvine, CA, USA). All RNA extracts were stored at −80 °C.

HEV RNA was detected using TaqMan reverse transcription quantitative PCR (RT-qPCR) targeting a 70 bp region within ORF3, as described by Jothikumar et al. [38]. To determine the RT-qPCR detection limit, serial dilutions were performed using the plasmid psK-HEV2 (GenBank accession no. AF444002.1), containing the full-length genome of the HEV Sarr-55 strain genotype 1 [39]. The established detection limit corresponded to a Ct of 37.5, equivalent to 2300 copies/mL. The reaction mixture (25 µL final volume) consisted of 12.5 µL of Tp2x reaction buffer, 0.5 µL of SuperScript III one-step RT-PCR Platinum Taq HiFi (Invitrogen by Thermo Fisher Scientific), 0.5 µL of each primer (10 µM), 0.4 µL of the probe (10 µM), and 5 µL of RNA template. Reverse transcription was carried out at 50 °C for 30 min, followed by denaturation at 95 °C for 10 min. DNA was amplified immediately using 50 PCR cycles at 95 °C (15 s), 55 °C (20 s), and 68 °C (30 s, with fluorescence acquisition).

For the sample that tested positive for HEV RNA by qRT-PCR (with a Ct value below 30), a region within ORF2/ORF3 was amplified using nested RT-PCR, as described by Inoue et al. [40]. The first round utilized HE361 (sense: 5-GCRGTGGTTTCTGGGGTGAC-3 and HE364 (antisense: 5-CTG GGM YTG GTC DCG CCA AG-3) primers, and the second round used HE366 (sense: 5-GYT GAT TCT CAG CCC TTC GC-3) and HE363 (antisense: 5-GMY TGG TCD CGC CAA GHGGA-3) primers. The size of the amplification product was 164 base pairs (bp) (nt 5302–5465) for the first-round PCR and 137 bp (nt 5325–5461) for the second-round PCR. The nucleotide numbers are in accordance with the HE-JA10 isolate (AB089824). The first round of amplification was performed using the SuperScript III One-Step RT-PCR System with Platinum Taq High Fidelity DNA Polymerase under the following conditions: 50 °C for 30 min; 94 °C for 2 min; 35 cycles of 94 °C for 15 s, 58 °C for 30 s, and 68 °C for 30 s; final extension at 68 °C for 5 min. The second round of PCR was performed using Platinum™ Taq DNA Polymerase (Thermo Fisher Scientific) under the following conditions: 94 °C for 2 min; 30 cycles of 94 °C for 30 s, 58 °C for 30 s, and 72 °C for 30 s; final extension at 72 °C for 7 min.

A second region within ORF2 was amplified using an additional nested RT-PCR protocol, as described by Fogeda et al. [41], using the primers HEV ORF 21F (sense: 5′-ACAGAATTRATTTCGTCGGC-3′) and HEV 21R (antisense: 5′-CCGRGTTTTACCYACCTTC-3′), generating an amplification product of 820 bp for the first round, and HEV ORF21FN (sense: 5′-GTCGTYTCRGCCAATGGC-3′) and HEVORF21RN (antisense: 5′GARAGCCAHARMACATCATT-3′), generating an amplification product of 220 bp for the second round. The first-round conditions were as follows: 50 °C for 30 min; 94 °C for 2 min; 39 cycles of 94 °C for 15 s, 48 °C for 30 s, and 68 °C for 30 s; final extension at 68 °C for 5 min. The second-round conditions were as follows: 94 °C for 2 min; 35 cycles of 94 °C for 30 s, 50 °C for 30 s, and 72 °C for 30 s; final extension at 72 °C for 7 min.

Amplicons of the expected size were purified and bidirectionally sequenced with 10 pmol of sense and antisense inner primers using the automatic sequencing service of Macrogen Inc. (Seoul, Republic of Korea). To monitor for potential PCR inhibition or inefficiency in viral recovery from wastewater, all samples were tested for Hepatitis A virus (HAV) RNA using a nested RT-PCR protocol, as described by Costa-Mattioli et al. [42].

### 2.6. Full-Length Genome Sequencing by Next-Generation Sequencing (NGS)

Complete genome sequencing was performed on samples with Ct values below 30 using next-generation sequencing. Viral RNA was extracted using the QIAamp Viral RNA Mini Kit (Qiagen, Hilden, Germany). Reverse transcription and PCR were performed using the SuperScript III or SuperScript IV One-Step RT-PCR System (Invitrogen, Carlsbad, CA, USA). SuperScript III was used to amplify fragments of up to 1000 bp, whereas SuperScript IV was employed for fragments larger than 1000 bp. The primers used to generate thirteen overlapping amplicons spanning the entire genome were as described by Chanmanee et al. [43] (Table 2). Amplicons were visualized by electrophoresis on a 2% (*w*/*v*) agarose gel. Approximately equimolar amounts of each amplicon were combined into a single pool to generate sequencing libraries.

Library preparation was performed using the Microbial Amplicon Prep Kit (iMAP) according to the Illumina Microbial Amplicon Reference Guide (Document # 200,039,808 v00), with IDT for Illumina-PCR Indexes set 1 (Illumina, San Diego, CA, USA), following the manufacturer’s instructions. Libraries were pooled, quantified (Qubit DNA HS, Thermo Fisher Scientific, Waltham, MA, USA), and sequenced on an iSeq 100 platform using a 300-cycle V2 kit with paired-end sequencing, with 10% PhiX control v3 and a loading concentration of 50 pM. Viral genome assembly was performed using the Genome Detective Virus Tool Version 2.24.0 “https://www.genomedetective.com/ (accessed on 30 August 2025)” [44]. The complete genome nucleotide sequence has been deposited in the GenBank database under accession number PX971288.

For all RT-PCR assays targeting HEV, the plasmid psK-HEV2 (GenBank accession no. AF444002.1), containing the full-length genome of the HEV Sarr-55 strain corresponding to genotype 1 [39], was used as a positive control.

### 2.7. Phylogenetic and Sequence Analyses

Sequence alignments were performed using MAFFT version 7 [45]. Phylogenetic analysis was performed using the maximum likelihood tree method with IQ-TREE version 3.1.1 “https://iqtree.github.io/ (accessed on 21 January 2026)” [46], incorporating 1000 bootstrap replicates. The best-fit substitution model was selected using ModelFinder within the IQ-TREE software. For the complete HEV genome tree, the General Time Reversible (GTR with +F, +I, +R5) model was applied. Reference sequences representing all major genotypes and subtypes, along with the most closely related sequences identified via BLAST analysis version 2.17.0, were included. Phylogenetic trees were visualized and annotated using iTOL version 7.4.2 “https://itol.embl.de/ (accessed on 21 January 2026)” [47].

### 2.8. HEV Subtyping

Nucleotide p-distance matrices were calculated for the full-length HEV genome against established reference sequences of subtypes 3a–3n and the putative subtype 3o. Analyses were conducted using MEGA 12 software (Pennsylvania State University, State College, PA, USA) [48], following the standard methods for HEV-3 subtyping classification [3,49].

### 2.9. Statistical Analysis

Statistical differences were assessed using the chi-square test with Yates correction or Fisher’s Exact test (when any expected value was less than 5). Statistical significance was set at a *p*-value of less than 0.05. All analyses were performed using Epi Info™ software version 7.2.7.0 (Centers for Disease Control and Prevention, Atlanta, GA, USA) [50].

## 3. Results

### 3.1. Prevalence of HEV in PLWH in Venezuela

**Figure 1:**
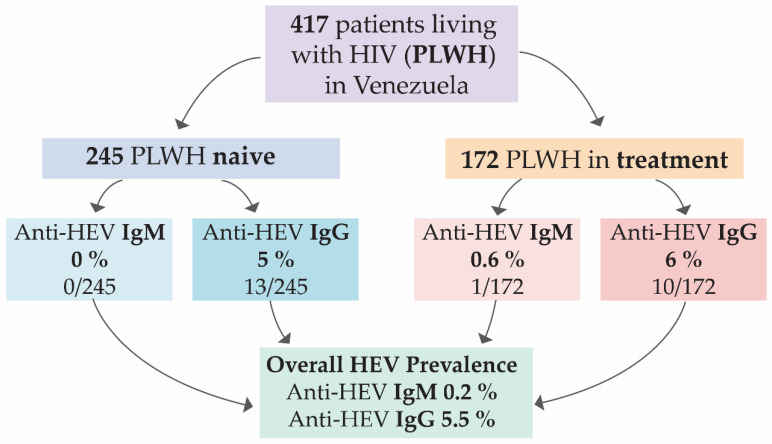
Prevalence of HEV according to the treatment status of individuals living with HIV in Venezuela.

Serological analyses for HEV were conducted on serum samples from 417 PLWH in Venezuela, collected between 2022 and 2025. Participants were stratified based on their antiretroviral therapy (ART) status as either naive (*n* = 245) or in treatment, with the latter representing individuals currently receiving or having a history of ART (*n* = 172) (Figure 1). The overall seroprevalence was found to be 0.2% (1/417) for anti-HEV IgM and 5.5% (23/417) for anti-HEV IgG. No statistically significant association was observed between ART status and seropositivity for either anti-HEV IgM (*p* = 0.412, Fisher’s Exact test) or anti-HEV IgG (*p* = 0.823, chi-square test) (Figure 1). Furthermore, HEV RNA was undetectable in any of the serum samples analyzed by RT-qPCR.

#### Prevalence of HEV in PLWH in Venezuela According to Age Group and Sex

The mean age of the participants was 36.6 years (mean ± 13.6 years; range, 10 months to 74 years), with a male predominance observed (70%; 291/417). This cohort included 33 children and adolescents (aged 10 months–17 years) and 18 pregnant women (Table 1).

Anti-HEV IgM seropositivity was identified in a single 42-year-old female, yielding a sex-specific prevalence of 0.8%, while no male participants were IgM-positive (Table 3). The overall anti-HEV IgG prevalence was 5.5% (representing 5% in males and 6% in females), with no statistically significant difference observed between sexes (IgM *p* = 0.302, Fisher’s Exact test; IgG *p* = 0.797, Yates’ correction) (Table 3). The highest IgG prevalence in males was observed in the 61–70 age group (15%), followed by the 21–30 age group (14%). In females, the highest prevalence was also identified in the 61–70 age group, followed by the 51–60 age group (Table 3). No significant association was found between seropositivity (IgM or IgG) and age when comparing individuals under and over 40 years of age (IgM: 0/258 vs. 1/159, *p* = 0.381, Fisher’s Exact test; IgG: 16/258 vs. 7/159, *p* = 0.575, Yates’ correction).

Among the group of children and adolescents, the anti-HEV IgG seroprevalence was 3% (1/33). All pregnant women (*n* = 18) were negative for anti-HEV IgM, anti-HEV IgG, and HEV RNA by RT-qPCR.

### 3.2. HEV Detection in Wastewater and Swine Fecal Samples

#### 3.2.1. Wastewater

Eighty-five wastewater samples, collected from several urban sectors of the Caracas metropolitan area over the same period (2022–2025) as the PLWH serum samples, were analyzed by RT-qPCR for HEV RNA. All samples tested negative (0/85). To rule out potential PCR inhibition or inefficient viral particle concentration during the pretreatment steps, the same RNA extracts were also tested for HAV RNA, another hepatitis virus transmitted via the fecal–oral route. HAV was detected in 28% (5/18) of the wastewater samples collected in 2024 and in 89% (24/27) of those collected in 2025, suggesting the absence of significant inhibitors in nucleic acid preparations.

#### 3.2.2. Swine

Sixty-seven swine fecal samples, sourced from one slaughterhouse and four family farms across four Venezuelan states (Miranda, Aragua, Guárico, and Bolívar), were screened by RT-qPCR. HEV RNA was detected in one isolate (designated HEV75, Ct = 19.9) from Bolívar State, yielding an overall prevalence of 1.5% (1/67). Sequence analysis of the partial ORF2 (820 bp) and ORF2/ORF3 (164 bp) regions confirmed its classification as genotype 3. Serum samples from individuals residing in the same region as the HEV75-positive swine, including pregnant women and individuals with direct swine contact, were found to be negative for HEV antibodies and RNA by RT-qPCR. It should be noted that the owners of the infected swine declined to provide personal samples for testing purposes.

#### 3.2.3. Whole-Genome Analysis of a Swine HEV Strain

The complete genome of the HEV75 isolate comprises 7207 nucleotides (excluding the poly(A) tail at the 3′ terminus) and encodes three major open reading frames: ORF1 (1703 aa, nt 4–5112), ORF2 (660 aa, nt 5147–7130), and ORF3 (114 aa, nt 5109–5453). The 5′ and 3′ untranslated regions (UTRs) span 25 and 76 nucleotides, respectively.

#### 3.2.4. Phylogenetic Analysis of the HEV75 Isolate

**Figure 2:**
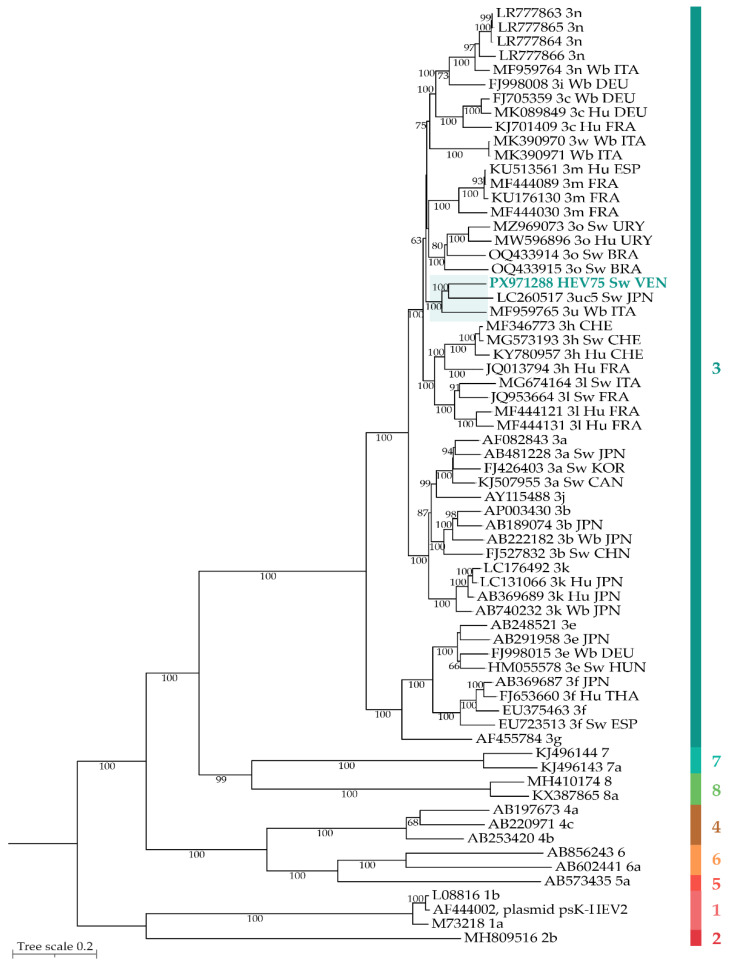
Phylogenetic tree of the complete HEV swine genome. Phylogeny was performed using the maximum likelihood method and the General Time Reversible (GTR) nucleotide substitution model. Differences in the rate of evolution between sites were modeled using empirical base frequencies (+F), with sites considered to be evolutionarily invariant (+I) and a free rate model with five categories (+R5). The number of each node corresponds to the bootstrap value obtained from 1000 replicates (bootstrap values greater than 70 are shown). Evolutionary analyses were performed on IQ-TREE. The right-hand bar represents the HEV genotypes, differentiated by color. Prototype strains from GenBank were designated by their accession numbers. The HEV isolate HEV75, obtained in this study, is highlighted in the color of the corresponding genotype. Each entry includes the accession number, HEV subtype, host (Hu, human; Sw, swine; Wb, wild boar), and country of origin of the isolate. ITA, Italy; DEU, Germany; FRA, France; ESP, Spain; URY, Uruguay; BRA, Brazil; JPN, Japan; CHE, Switzerland; KOR, Republic of Korea; CAN, Canada; CHN, China; HUN, Hungary; THA, Thailand; VEN, Venezuela.

Phylogenetic analysis positioned HEV75 within the HEV genotype 3 clade. The HEV75 isolate formed a robust cluster (bootstrap value: 100%) with two complete genome sequences: a swine isolate from Japan (LC260517) [51] and a wild boar isolate from Italy (MF959765) [52], both of which remained unclassified into any of the subtypes assigned thus far within genotype 3 (Figure 2). This unassignable subtype 3 cluster exhibits nucleotide identities of 87.14–87.30% and amino acid identities of 97.4–98.1% for ORF1, 97.1–98.8% for ORF2, and 61.2–84.7% for ORF3. This cluster may represent a novel subtype, tentatively designated as 3p, following the subtype 3o previously proposed by Cancela et al. [11].

#### 3.2.5. HEV75 Subtype

To further characterize the HEV75 complete genome at the subtype level, pairwise nucleotide p-distance matrices were calculated. The HEV75 complete genome was compared with representative sequences of each subtype (3a-n and putative 3o, Table 4). The analysis revealed p-distance values ranging from 0.140 to 0.193 for HEV75 relative to reference subtypes 3a–3o. In contrast, the distances to the two sequences classified as unassignable subtype 3 (LC260517 [51] from Japan and MF959765 [52] from Italy) were lower, ranging from 0.128 to 0.129. This genetic proximity supports the clustering observed in the phylogenetic tree and suggests that HEV75, together with these unassignable subtype 3 sequences, may constitute a distinct subtype within genotype 3.

## 4. Discussion

HEV is primarily transmitted via the fecal–oral route and, to a lesser extent, through blood transfusions, organ transplants, and vertical transmission [2,12,13,14,15,16,17,18]. Although HEV infection typically presents as an acute, self-limiting illness, co-infection with HIV can lead to chronic infection, accelerating the progression to liver fibrosis and cirrhosis [15,16,17]. In pregnant women, HEV infection may induce acute liver failure, with mortality rates reaching up to 30% during the third trimester [18,19,20]. During acute infection, HEV RNA is detectable in the blood for two to six weeks post-infection, whereas fecal shedding may persist for up to two weeks longer. IgM antibodies appear at 3–4 weeks and persist for 4–6 months, while IgG antibodies can last for several years [53,54]. A similar serological pattern is observed in immunocompromised patients; however, during chronic infection, persistent viremia and fecal shedding of HEV RNA are frequent [15,53,54].

In this study, the seroprevalence of HEV among PLWH in Venezuela was found to be 0.2% for IgM and 5.5% for IgG antibodies. The IgM prevalence was lower than that reported in Venezuelan patients with acute hepatitis (30%) [36] and patients with HIV in Brazil (1.4%) [55], but similar to the rate found in vulnerable populations in Central Brazil (0.22%) [56]. The heterogeneity of these study cohorts precludes a direct epidemiological comparison. The IgG seroprevalence was lower than that reported for Brazilian PLWH (10%) [55], but comparable to rates in PLWH from Argentina (5.2%) [57] and northeastern Brazil (4.1%) [58]. Serological HEV test performance can vary significantly depending, for example, on the commercial supplier and antigen composition, factors that are particularly critical when assessing seroprevalence in immunocompromised populations. A comprehensive meta-analysis evaluating the diagnostic accuracy of HEV antibody tests confirmed that among immunocompromised patients, the pooled sensitivity and specificity for IgM assays were 86% and 99%, respectively [59]. In contrast, under the same evaluation conditions using PCR as the reference standard, IgG assays exhibited substantially lower sensitivity (ranging from 15% to 65%) than IgM assays [59]. In this context, the Adaltis EIAgen assay utilized in the present study was previously assessed in a cohort of immunocompromised patients with acute HEV infection. In that evaluation, the IgM assay demonstrated adequate sensitivity within this population, whereas the IgG assay showed reduced sensitivity, despite maintaining high specificity [60]. Consequently, the 5.5% IgG seroprevalence observed in this cohort of Venezuelan PLWH may underestimate the actual rate of previous HEV exposure.

No active HEV infection was detected in the human cohort, as HEV RNA was found to be undetectable by RT-qPCR in all serum samples from PLWH. The absence of viremia in HEV-seropositive PLWH has been reported in other Latin American studies [55,56,57,58]. However, it cannot be ruled out that this discrepancy could be influenced by the patient’s immune status, infection phase (e.g., convalescence), HEV genotype, or the sensitivity of the diagnostic assays [15,59,61]. The combined use of serological and molecular tests remains the optimal approach for HEV diagnosis [59,61]. The seropositivity observed in our cohort, even in the absence of RNA, indicates prior viral exposure in this immunocompromised population and justifies continued clinical surveillance. The reinforcement of hygiene protocols, guaranteed access to potable water, and adequate sanitation constitute critical public health interventions.

Epidemiological data on HEV in pediatric and adolescent populations living with HIV are scarce. The anti-HEV IgG prevalence of 3% (1/33) in this subgroup was lower than that in immunocompromised children from Argentina (6.9%) [62] and young patients with acute hepatitis in Venezuela (27–38%) [36]. The sole seropositive case was an 8-year-old girl who had contact with rabbits, chickens, and tortoises. While rabbits are known reservoirs for HEV-3ra, chickens and tortoises are not established reservoirs for HEV [2,3]. Although active infection was not detected, IgG seropositivity indicated prior, possibly asymptomatic, exposure, suggesting that HEV transmission occurs from an early age in Venezuela, with zoonotic contact representing a plausible risk factor.

HEV infection during pregnancy is associated with significant maternal mortality, preterm labor, and vertical transmission [2,18,19,20]. Data from the Americas remain scarce. In Mexico, a region considered endemic, the prevalence among pregnant women ranges from 0.22% to 5.7% [63,64]. In Argentina, the anti-HEV IgG seroprevalence was higher in pregnant women (8.4%) than in non-pregnant women (2.6%) [65], whereas a study in Southern Brazil reported a prevalence of 19% [66]. In Venezuela, the most recent data from 1994 indicated an anti-HEV prevalence of 1.9% in pregnant women from *Maternidad Concepción Palacios* and 1.3% in those from a private clinic in the capital [35]. Notably, all these reports correspond to immunocompetent populations. Our investigation found no serological or molecular evidence of HEV among pregnant women living with HIV, constituting the first report on this specific demographic in Venezuela and highlighting the need to integrate HEV screening into routine antenatal care, irrespective of HIV serostatus. However, the number of tested pregnant women was insufficient to allow for any definitive comparison.

Wastewater-based epidemiology (WBE) constitutes a robust tool for monitoring community-level pathogen circulation. In South America, environmental detection has been reported exclusively for genotype 3 [11,30,31,32,33,34]. Our analysis of wastewater collected from various sites in the Capital District between 2022 and 2025 yielded no detection of HEV RNA by RT-qPCR. This finding is consistent with the surveillance of river water in southern Brazil [34], but contrasts with higher reported detection rates in wastewater from Colombia (16.7%) [32], Argentina (22.5%) [31], and Uruguay (10.87%) [11]. To our knowledge, this represents the first survey for HEV in Venezuelan wastewater. The absence of environmental detection correlates with the low seroprevalence observed in our PLWH cohort, primarily recruited from the capital region, and underscores the necessity of extending environmental surveillance nationally. As an internal control for nucleic acid recovery and PCR efficiency, HAV RNA was detected in 64% of wastewater samples from 2024 to 2025, a rate consistent with reported ranges for untreated wastewater in Latin America (2.8–70.9%) [67].

Human infections are caused by HEV genotypes 1, 2, 3, 4, and 7, with genotypes 3 and 4 maintained in domestic pigs and wild boars as principal reservoirs [8,9,15]. Domestic swine constitute a major HEV reservoir, with global molecular prevalence estimates of approximately 13% [2,68]. In this study, HEV RNA was detected in 1.5% (1/67) of swine fecal samples from four Venezuelan states. The reliable detection of HEV RNA in swine fecal samples by RT-qPCR can be challenging due to the presence of inhibitory substances, a high microbial background, and variable levels of RNA degradation. These factors may lead to reduced assay sensitivity or false-negative results [69]. Consequently, the inclusion of appropriate internal amplification controls and the validation of RNA extraction methods to ensure effective inhibitor removal are essential to ensure the accuracy of molecular diagnostics in swine fecal specimens [69]. However, internal controls were not included in the present study. Therefore, the possibility that the low prevalence observed may be attributable to the presence of RT-qPCR inhibitors in the analyzed samples cannot be entirely excluded. Additionally, the lack of serological prevalence data for these swine represents a limitation of this study.

HEV transmission among swine primarily occurs via the fecal–oral route through direct contact or environmental contamination. The virus exhibits significant environmental persistence, remaining infectious in feces and on surfaces for days [70]. Furthermore, HEV has been detected in manure from grow–finish pig farms [71]. In this study, the HEV-positive specimen was a single domesticated animal, the only one present on the property, and was fed with food scraps. Although the fecal sample was collected immediately after defecation to avoid soil contact, the possibility of environmental contamination from the floor or other fomites cannot be definitively ruled out. Nevertheless, the finding of HEV in a swine from a rural community in Bolívar state is the first reported detection of HEV in Venezuelan swine and suggests the need for expanded surveillance of reservoirs (including wastewater) in this and other states, particularly in rural areas. No serological or molecular evidence of HEV was found in a limited human cohort from the same community, including pregnant women and individuals in contact with swine. However, direct exposure assessment was limited, as the owners of the viremic swine declined to provide samples.

Phylogenetic analysis of the complete genome of the swine isolate (HEV75) confirmed its classification as HEV genotype 3, the dominant zoonotic genotype in Latin America [23,24,25,26,27,28]. HEV-3 is partitioned into multiple assigned subtypes (a-o, 3ra) [3] with provisionally designated unassigned strains (e.g., 3u, 3w) [72]. The HEV75 isolate formed a monophyletic cluster with two unassignable subtype 3 sequences from Japan (LC260517) [51] and Italy (MF959765) [52]. According to the proposed criteria, a cut-off value of 0.093 nucleotide substitutions per site is applied to define subtypes [49]. The pairwise distances between the HEV75 isolate and reference sequences for established subtypes (3a-3n, and putative 3o) ranged from 0.140 to 0.193, exceeding this threshold. Notably, the distances to unassignable subtype 3 sequences (LC260517 and MF959765) were lower (0.128–0.129), indicating a closer phylogenetic affinity. As the pairwise distances within this cluster (HEV75, LC260517, MF959765) all exceeded the 0.093 cut-off relative to other subtypes, and the cluster comprised three complete, epidemiologically unrelated genomes, thereby fulfilling the proposed criteria for new subtype designation [10], we propose that it represents a novel subtype within HEV-3, designated as 3p.

In Venezuela, human infections with HEV genotypes 1 and 3 have been documented, with genotype 1 being more prevalent [36]. The detection of HEV-3 in swine confirmed the presence of potential zoonotic reservoirs. Although our data suggest a currently low prevalence, HEV should be included in the differential diagnosis for at-risk groups, including PLWH, pregnant women, and individuals with occupational or domestic swine exposure. The implementation of integrated One Health surveillance spanning human, animal (swine), and environmental (wastewater) compartments is recommended to elucidate national epidemiology and inform prevention strategies.

## 5. Conclusions

This study provides serological evidence of prior HEV exposure, albeit at a low prevalence, among PLWH in Venezuela, including pediatric populations. Surveillance of potential reservoirs revealed the absence of HEV RNA in wastewater from the capital region but identified an active HEV-3 infection in swine from Bolívar State. To our knowledge, this constitutes the first molecular confirmation of HEV genotype 3 circulation in a Venezuelan swine, establishing a potential zoonotic reservoir with public health implications. Furthermore, phylogenetic analysis of the complete viral genome suggests that it belongs to a novel subtype. This genome represents the third reported for this unassignable subtype 3 cluster (alongside sequences from Japan and Italy), satisfying the proposed criterion of three phylogenetically distinct and epidemiologically unrelated genomes required for new subtype definition, tentatively designated as 3p.

## Figures and Tables

**Table 1:** Study population.

Population Type	PLWH	HIV Negative
Children and adolescents ^1^	33	-
18 to over 70 years old	366	-
Pregnant women	18	6
Close contact with swine	-	12
Total	417 (70% males)	18

^1^ Aged between 10 months and 17 years old.

**Table 2:** Primers used for HEV whole-genome-sequence amplification.

Region	Primer	Primer Sequence ^1^	Position	Product Size (bp)
5′UTR/ORF1 (1)	HE5′ HE52	5′-GTC GAT GCC ATG GAG GCC-3′ 5′-CCG AGG GCC AAA GGT CAT G-3′	1-18 525-543	543
ORF1 (2)	HE01 HE02	5′-AAG GCT CCT GGC ATT ACT ACT-3′ 5′-AAR AGC ATR AGC CGR TCT CA-3′	48-70 984-1003	955
ORF1 (3)	HE741F HE1895Rst	5′-TGG ATC CGC ACC ACT AAA ATA-3′ 5′-CGG TRC AAT CCA RGC CAT TA-3′	741-761 1895-1914	1173
ORF1 (4)	HE1498Fst HE2293Rst	5′-TGG TGG GTG ACT GTG GCC AT -3′ 5′-TGG TGT RGG CGG CTT ACG G -3′	1498-1517 2357-2375	877
ORF1 (5)	HE1990F HE3054Rst	5′-ACA ATA GGT TCA CCC AGC GCC-3′ 5′-GTC GCC AAC TAT TGC GGA GCT C-3′	1990-2010 2985-3006	1085
ORF1 (6)	FfrR4st FfR4n’	5′-GTC GAT GCC ATG GAG GCC-3′ 5′-CCG AGG GCC AAA GGT CAT G-3′	2795-2815 3783-3802	1007
ORF1 (7)	HE3560Fst HE4497R	5′-TGC GCA TGC TAT CGT TGC ACT-3′ 5′-GGC ATG CCA CAY TCC TCC AT-3′	3560-3580 4497-4516	956
ORF1 (8)	HE4263Fst HE5058R	5′-GCA TAT CGG CTT GGA GTA AGA C-3′ 5′-AGT GGG CCT TTC CAT CAG CAA T-3′	4263-4285 5058-5079	816
ORF1/ORF2 (9)	HE4974F HE5631Rst	5′-CGC AGG TTT GTG TTG ATG TTG T-3′ 5′-GGG GCG GCA TAC AAG ACA AGA-3′	4974-4996 5631-5651	677
ORF2 (10)	HE5204F HE6159Rst	5′-TCT TCG TGC TTC TGC CTA TGC TG-3′ 5′-CCA CGA CGC AAA CGG TGT-3′	5204-5226 6159-6176	972
ORF2 (11)	ORF2F2 ORF2R2	5′-CHA TYT CTA TYT CYT TYT GGC C-3′ 5′-GTA GTC TGR TCA TAY TCA GCV GC-3′	5890-5912 6524-6499	955
ORF2/3′UTR (12)	HE6209Fst HE3prime	5′-CAG CCA CAC GTT TYA TGA AGG A-3′ 5′-TTT TTT TTT TTT TTT TTT CCA GGG AGC G-3′	6209-6203 7220-7245	1032
ORF1/3′UTR (13)	HE4263Fst HE3prime	5′-GCA TAT CGG CTT GGA GTA AGA C-3′ 5′-TTT TTT TTT TTT TTT TTT CCA GGG AGC G-3′	4263-4285 7220-7245	2982

^1^ All these primers were described by Chanmanee et al. [43].

**Table 3:** Prevalence of IgM and IgG antibodies against HEV in PLWH, by sex and age.

		Positive HEV Serological Markers						
		Ig M			Ig G			
Age (Years)	F	%	M	%	F	%	M	%
0–10	0/8	0	0/4	0	1/8	12.5	0/4	0
11–20	0/17	0	0/20	0	0/17	0	0/20	0
21–30	0/20	0	0/80	0	1/20	5	11/80	14
31–40	0/30	0	0/79	0	2/30	7	1/79	1
41–50	**1/34**	**3**	0/57	0	2/34	6	0/57	0
51–60	0/9	0	0/38	0	1/9	11	1/38	3
61–70	0/7	0	0/13	0	**1/7**	**14**	**2/13**	**15**
Over 70	0/1	0	0/0	0	0/1	0	0/0	0
Total	1/126	0.8	0/291	0	8/126	6	15/291	5
Statistical difference	*p* = 0.302				*p* = 0.797			

F, female; M, male. The higher prevalence by sex, age group, and serological marker is highlighted in bold.

**Table 4:** Nucleotide p-distance between the complete genome of the HEV75 isolate and reference sequences of HEV-3 subtypes proposed in 2020 by Smith et al. [3] and deposited in the NCBI database.

Accession Number of the Complete Genome	Subtype	p-Distance ^1^
AF082843 ^2^, KJ507955, AB481228, FJ426403	3a	0.151–0.157
AP003430 ^2^, AB222182, AB189074, FJ526832	3b	0.156–0.162
FJ705359 ^2^, MK089849, KJ701409	3c	0.144–0.145
AB248521 ^2^, AB291958, FJ998015, HM055578	3e	0.183–0.186
AB369687 ^2^, EU375463, FJ653660, EU723513	3f	0.189–0.193
AF455784 ^2^	3g	0.185
JQ013794 ^2^, MF346773, MG573193, KY780957	3h	0.140–0.148
FJ998008 ^2^	3i	0.141
AY115488 ^2^	3j	0.164
AB369689 ^2^, LC176492, LC131066, AB740232	3k	0.155–0.158
JQ953664 ^2^, MG674164, MF444121, MF444131	3l	0.142–0.148
KU513561 ^2^, MF444089, KU176130, MF444030	3m	0.143–0.145
MF959764 ^2^, LR777863-66	3n	0.141–0.147
OQ433914 ^2^, OQ433915 ^2^, M7969073, MW596896	Putative 3o [11]	0.140–0.149
LC260517 [51], MF959765 [52]	Unassigned	0.123–0.129

^1^ Minimum and maximum values are shown. ^2^ References HEV complete genome sequences [3]. No complete genome is available for the 3d subtype; therefore, this subtype was not included.

## Data Availability

The complete HEV genome sequence has been deposited in GenBank under accession number PX971288.

## Abbreviations

The following abbreviations are used in this manuscript:

HEV	Hepatitis E virus
HIV	Human immunodeficiency virus
PLWH	People living with HIV
WBE	Wastewater-based epidemiology
ELISA	Enzyme-linked immunosorbent assay
HAV	Hepatitis A virus

## References

[1] World Health Organization (2025). Hepatitis E Fact Sheet. https://www.who.int/news-room/fact-sheets/detail/hepatitis-e.

[2] Songtanin B., Molehin A.J., Brittan K., Manatsathit W., Nugent K. (2023). Hepatitis E Virus Infections: Epidemiology, Genetic Diversity, and Clinical Considerations. Viruses.

[3] Smith D.B., Izopet J., Nicot F., Simmonds P., Jameel S., Meng X.J., Norder H., Okamoto H., van der Poel W.H.M., Reuter G. (2020). Update: Proposed reference sequences for subtypes of hepatitis E virus (species Orthohepevirus A). J. Gen. Virol..

[4] Shafat Z., Ahmed A., Parvez M.K., Parveen S. (2021). Sequence to structure analysis of the ORF4 protein from Hepatitis E virus. Bioinformation.

[5] Kenney S.P., Meng X.J. (2019). Hepatitis E Virus Genome Structure and Replication Strategy. Cold Spring Harb. Perspect. Med..

[6] Purdy M.A., Harrison T.J., Jameel S., Meng X.J., Okamoto H., Van der Poel W.H.M., Smith D.B., Ictv Report Consortium (2017). ICTV Virus Taxonomy Profile: *Hepeviridae*. J. Gen. Virol..

[7] Primadharsini P.P., Nagashima S., Okamoto H. (2019). Genetic Variability and Evolution of Hepatitis E Virus. Viruses.

[8] Spahr C., Knauf-Witzens T., Vahlenkamp T., Ulrich R.G., Johne R. (2018). Hepatitis E virus and related viruses in wild, domestic and zoo animals: A review. Zoonoses Public Health.

[9] Tene S.D., Diouara A.A.M., Sané S., Coundoul S. (2025). Hepatitis E Virus (HEV) Infection in the Context of the One Health Approach: A Systematic Review. Pathogens.

[10] Smith D.B., Simmonds P., Izopet J., Oliveira-Filho E.F., Ulrich R.G., Johne R., Koenig M., Jameel S., Harrison T.J., Meng X.J. (2016). Proposed reference sequences for hepatitis E virus subtypes. J. Gen. Virol..

[11] Cancela F., Icasuriaga R., Cuevas S., Hergatacorzian V., Olivera M., Panzera Y., Pérez R., López J., Borzacconi L., González E. (2023). Epidemiology Update of Hepatitis E Virus (HEV) in Uruguay: Subtyping, Environmental Surveillance and Zoonotic Transmission. Viruses.

[12] Raji Y.E., Toung O.P., Taib N.M., Sekawi Z.B. (2022). Hepatitis E Virus: An emerging enigmatic and underestimated pathogen. Saudi J. Biol. Sci..

[13] Kamar N., Garrouste C., Haagsma E.B., Garrigue V., Pischke S., Chauvet C., Dumortier J., Cannesson A., Cassuto-Viguier E., Thervet E. (2011). Factors associated with chronic hepatitis in patients with hepatitis E virus infection who have received solid organ transplants. Gastroenterology.

[14] Lee G.H., Tan B.H., Teo E.C., Lim S.G., Dan Y.Y., Wee A., Aw P.P., Zhu Y., Hibberd M.L., Tan C.K. (2016). Chronic Infection with Camelid Hepatitis E Virus in a Liver Transplant Recipient Who Regularly Consumes Camel Meat and Milk. Gastroenterology.

[15] Lhomme S., Marion O., Abravanel F., Izopet J., Kamar N. (2020). Clinical Manifestations, Pathogenesis and Treatment of Hepatitis E Virus Infections. J. Clin. Med..

[16] Philippart M., Vanwolleghem T., Dahlqvist G. (2025). Chronic hepatitis E in immunosuppressed patients: A comprehensive review of the literature. Acta Gastro-Enterol. Belg..

[17] Philippart M., Peeters M., Lamoral S., Piessevaux H., Badii M.C., Bailly S., Poiré X., Kanaan N., Devresse A., Kabamba B. (2025). Chronic hepatitis E and hepatitis E seroprevalence in immunosuppressed patients: A prospective study. Transl. Gastroenterol. Hepatol..

[18] Krain L.J., Atwell J.E., Nelson K.E., Labrique A.B. (2014). Fetal and Neonatal Health Consequences of Vertically Transmitted Hepatitis E Virus Infection. Am. J. Trop. Med. Hyg..

[19] Wu C., Wu X., Xia J. (2020). Hepatitis E virus infection during pregnancy. Virol. J..

[20] Haase J.A., Schlienkamp S., Ring J.J., Steinmann E. (2025). Transmission Patterns of Hepatitis E Virus. Curr. Opin. Virol..

[21] Fousekis F.S., Mitselos I.V., Christodoulou D.K. (2020). Extrahepatic manifestations of hepatitis E virus: An overview. Clin. Mol. Hepatol..

[22] Jha A.K., Kumar G., Dayal V.M., Ranjan A., Suchismita A. (2021). Neurological manifestations of hepatitis E virus infection: An overview. World J. Gastroenterol..

[23] Fernández Villalobos N.V., Kessel B., Rodiah I., Ott J.J., Lange B., Krause G. (2022). Seroprevalence of hepatitis E virus infection in the Americas: Estimates from a systematic review and meta-analysis. PLoS ONE.

[24] Pisano M.B., Martinez-Wassaf M.G., Mirazo S., Fantilli A., Arbiza J., Debes J.D., Ré V.E. (2018). Hepatitis E virus in South America: The current scenario. Liver Int..

[25] Pisano M.B., Mirazo S., Re V.E. (2020). Hepatitis E Virus Infection: Is It Really a Problem in Latin America?. Clin. Liver Dis..

[26] Magri M.C., Manchiero C., Dantas B.P., Bernardo W.M., Abdala E., Tengan F.M. (2025). Prevalence of hepatitis E in Latin America and the Caribbean: A systematic review and meta-analysis. Public Health.

[27] de Oliveira J.M., dos Santos D.R.L., Pinto M.A. (2023). Hepatitis E Virus Research in Brazil: Looking Back and Forwards. Viruses.

[28] Williman M.M., Colina S.E., Di Cola G., Ozaeta D.S., Carpinetti B.N., Pisano M.B., Ré V.E., Serena M.S., Echeverría M.G., Metz G.E. (2026). Evidence of Wild Boars as a Reservoir of Zoonotic Hepatitis E Virus Genotype 3: Implications for Public Health in Argentina. Pathogens.

[29] Moraes D.F.D.S.D., Mesquita J.R., Dutra V., Nascimento M.S.J. (2021). Systematic Review of Hepatitis E Virus in Brazil: A One-Health Approach of the Human-Animal-Environment Triad. Animals.

[30] Takuissu G.R., Kenmoe S., Ndip L., Ebogo-Belobo J.T., Kengne-Ndé C., Mbaga D.S., Bowo-Ngandji A., Oyono M.G., Kenfack-Momo R., Tchatchouang S. (2022). Hepatitis E Virus in Water Environments: A Systematic Review and Meta-analysis. Food Environ. Virol..

[31] Lo Castro I., Espul C., de Paula V.S., Altabert N.R., Gonzalez J.E., Lago B.V., Villar L.M. (2023). High prevalence of hepatitis A and E viruses in environmental and clinical samples from West Argentina. Braz. J. Infect. Dis..

[32] Baez P.A., Lopez M.C., Duque-Jaramillo A., Pelaez D., Molina F., Navas M.C. (2017). First evidence of the Hepatitis E virus in environmental waters in Colombia. PLoS ONE.

[33] Pisano M.B., Lugo B.C., Poma R., Cristobal H.A., Raskovsky V., MartinezWassaf M.G., Rajal V.B., Re V.E. (2018). Environmental hepatitis E virus detection supported by serological evidence in the northwest of Argentina. Trans. R. Soc. Trop. Med. Hyg..

[34] Heldt F.H., Staggmeier R., Gularte J.S., Demoliner M., Henzel A., Spilki F.R. (2016). Hepatitis E Virus in SurfaceWater, Sediments, and Pork Products Marketed in Southern Brazil. Food Environ. Virol..

[35] Pujol F.H., Favorov M.O., Marcano T., Esté J.A., Magris M., Liprandi F., Khudyakov Y.E., Khudyakova N.S., Fields H.A. (1994). Prevalence of antibodies against hepatitis E virus among urban and rural populations in Venezuela. J. Med. Virol..

[36] Gutiérrez García C., Sánchez D., Villalba M.C., Pujol F.H., de Los Ángeles Rodríguez Lay L., Pinto B., Chacón E.P., Guzmán M.G. (2012). Molecular characterization of hepatitis E virus in patients with acute hepatitis in Venezuela. J. Med. Virol..

[37] Bastardo-Méndez M., Rangel H.R., Pujol F.H., Grillet M.E., Jaspe R.C., Malaver N., Rodríguez M., Zamora-Figueroa A. (2024). Detection of SARS-CoV-2 in wastewater as an earlier predictor of COVID-19 epidemic peaks in Venezuela. Sci. Rep..

[38] Jothikumar N., Cromeans T.L., Robertson B.H., Meng X.J., Hill V.R. (2006). A broadly reactive one-step real-time RT-PCR assay for rapid and sensitive detection of hepatitis E virus. J. Virol. Methods.

[39] Emerson S.U., Zhang M., Meng X.J., Nguyen H., St Claire M., Govindarajan S., Huang Y.K., Purcell R.H. (2001). Recombinant hepatitis E virus genomes infectious for primates: Importance of capping and discovery of a cis-reactive element. Proc. Natl. Acad. Sci. USA.

[40] Inoue J., Takahashi M., Yazaki Y., Tsuda F., Okamoto H. (2006). Development and validation of an improved RT-PCR assay with nested universal primers for detection of hepatitis E virus strains with significant sequence divergence. J. Virol. Methods.

[41] Fogeda M., Avellón A., Cilla C.G., Echevarría J.M. (2009). Imported and autochthonous hepatitis E virus strains in Spain. J. Med. Virol..

[42] Costa-Mattioli M., Cristina J., Romero H., Perez-Bercof R., Casane D., Colina R., Garcia L., Vega I., Glikman G., Romanowsky V. (2002). Molecular evolution of hepatitis A virus: A new classification based on the complete VP1 protein. J. Virol..

[43] Chanmanee T., Ajawatanawong P., Louisirirotchanakul S., Chotiyaputta W., Chainuvati S., Wongprompitak P. (2020). Phylogenetic analysis of two new complete genomes of the hepatitis E virus (HEV) genotype 3 from Thailand. Mol. Biol. Rep..

[44] Vilsker M., Moosa Y., Nooij S., Fonseca V., Ghysens Y., Dumon K., Pauwels R., Alcantara L.C., Vanden Eynden E., Vandamme A.M. (2019). Genome Detective: An automated system for virus identification from high-throughput sequencing data. Bioinformatics.

[45] Katoh K., Standley D.M. (2013). MAFFT multiple sequence alignment software version 7: Improvements in performance and usability. Mol. Biol. Evol..

[46] Wong T., Ly-Trong N., Ren H., Banos H., Roger A.J., Susko E., Bielow C., De Maio N., Goldman N., Hahn M.W. (2025). IQ-TREE 3: Phylogenomic Inference Software using Complex Evolutionary Models. Submitted. https://ecoevorxiv.org/repository/view/8916/.

[47] Letunic I., Bork P. (2024). Interactive Tree of Life (iTOL) v6: Recent updates to the phylogenetic tree display and annotation tool. Nucleic Acids Res..

[48] Kumar S., Stecher G., Suleski M., Sanderford M., Sharma S., Tamura K. (2024). MEGA12: Molecular Evolutionary Genetics Analysis version 12 for adaptive and green computing. Mol. Biol. Evol..

[49] Nicot F., Dimeglio C., Migueres M., Jeanne N., Latour J., Abravanel F., Ranger N., Harter A., Dubois M., Lameiras S. (2021). Classification of the Zoonotic Hepatitis E Virus Genotype 3 Into Distinct Subgenotypes. Front. Microbiol..

[50] Centers for Disease Control and Prevention (2025). Epi Info™.

[51] Primadharsini P.P., Miyake M., Kunita S., Nishizawa T., Takahashi M., Nagashima S., Tanggis, Ohnishi H., Kobayashi T., Nishiyama T. (2017). Full-length genome of a novel genotype 3 hepatitis E virus strain obtained from domestic pigs in Japan. Virus Res..

[52] De Sabato L., Di Bartolo I., Lapa D., Capobianchi M.R., Garbuglia A.R. (2020). Molecular Characterization of HEV Genotype 3 in Italy at Human/Animal Interface. Front. Microbiol..

[53] Velavan T.P., Pallerla S.R., Johne R., Todt D., Steinmann E., Schemmerer M., Wenzel J.J., Hofmann J., Shih J.W.K., Wedemeyer H. (2021). Hepatitis E: An update on One Health and clinical medicine. Liver Int..

[54] Aslan A.T., Balaban H.Y. (2020). Hepatitis E virus: Epidemiology, diagnosis, clinical manifestations, and treatment. World J. Gastroenterol..

[55] Ferreira A.C., Gomes-Gouvêa M.S., Lisboa-Neto G., Mendes-Correa M.C.J., Picone C.M., Salles N.A., Mendrone-Junior A., Carrilho F.J., Pinho J.R.R. (2018). Serological and molecular markers of hepatitis E virus infection in HIV-infected patients in Brazil. Arch. Virol..

[56] Teles S.A., Caetano K.A.A., Carneiro M.A.D.S., Villar L.M., Stacciarini J.M., Martins R.M.B. (2023). Hepatitis E Prevalence in Vulnerable Populations in Goiânia, Central Brazil. Viruses.

[57] Acosta J., Galimberti A., Marziali F., Bessone F., Costaguta A., Águila D., Lupo S., Rocha De Lima B., Tanno H., Reggiardo V. (2023). P-50 Seroprevalence of Hepatitis E Virus in HIV-infected patients from Rosario, Santa Fe. Ann. Hepatol..

[58] Bezerra L.A., de Oliveira-Filho E.F., Júnior J.V.J.S., Morais V.M.S., Gonçales J.P., da Silva D.M., Duarte Coêlho M.R.C. (2019). Risk analysis and seroprevalence of HEV in people living with HIV/AIDS in Brazil. Acta Trop..

[59] Mirzaev U.K., Yoshinaga Y., Baynazarov M., Ouoba S., Ko K., Phyo Z., Chhoung C., Akuffo G.A., Sugiyama A., Akita T. (2025). Diagnostic accuracy of hepatitis E virus antibody tests: A comprehensive meta-analysis. Hepatol. Res..

[60] Abravanel F., Lhomme S., Chapuy-Regaud S., Mansuy J.M., Muscari F., Sallusto F., Rostaing L., Kamar N., Izopet J. (2013). Performance of a new rapid test for detecting anti-hepatitis E virus immunoglobulin M in immunocompromised patients. J. Clin. Virol..

[61] Zhang Q., Zong X., Li D., Lin J., Li L. (2020). Performance Evaluation of Different Commercial Serological Kits for Diagnosis of Acute Hepatitis E Viral Infection. Pol. J. Microbiol..

[62] Acosta J., Costaguta G., Gardiol D., Álvarez F., Costaguta A., Cavatorta A.L. (2025). Hepatitis E virus in immunocompromised children in Argentina: First report from a high-risk group. Virol. J..

[63] García-Romero C.S., Guzmán C., Martínez-Ibarra A., Cervantes A., Cerbón M. (2024). Viral Hepatitis in Pregnant Mexican Women: Its Impact in Mother-Child Binomial Health and the Strategies for Its Eradication. Pathogens.

[64] Alvarado-Esquivel C., Sánchez-Anguiano L.F., Hernández-Tinoco J. (2014). Hepatitis E virus exposure in pregnant women in rural Durango, Mexico. Ann. Hepatol..

[65] Tissera G., Lardizabal M.C., Torres S.B., Fantilli A.C., Martínez Wassaf M.G., Venezuela F., Capra R., Balderramo D.C., Travella C., Ré V.E. (2020). Hepatitis E virus infection in pregnant women, Argentina. BMC Infect. Dis..

[66] Hardtke S., Rocco R., Ogata J., Braga S., Barbosa M., Wranke A., Doi E., da Cunha D., Maluf E., Wedemeyer H. (2018). Risk factors and seroprevalence of hepatitis E evaluated in frozen-serum samples (2002–2003) of pregnant women compared with female blood donors in a Southern region of Brazil. J. Med. Virol..

[67] Ré V.E., Ridruejo E., Fantilli A.C., Moutinho B.D., Pisano M.B., Pessoa M.G. (2024). Hepatitis A in Latin America: The current scenario. Rev. Med. Virol..

[68] Li P., Ji Y., Li Y., Ma Z., Pan Q. (2021). Estimating the global prevalence of hepatitis E virus in swine and pork products. One Health.

[69] Hoffmann B., Hoffmann D., Henritzi D., Beer M., Harder T.C. (2020). Inhibition monitoring in veterinary molecular testing. J. Vet. Diagn. Investig..

[70] Meester M., Valenzuela Agüí C., Tobias T.J., Hakze van der Honing R.W., Guinat C., Bouwknegt M., du Plessis L., Fischer E.A.J., Spaninks M., Stadler T. (2025). Zoonotic hepatitis E virus spreads through environmental routes in pig herds—A phylodynamic analysis. PLoS Pathog..

[71] Souza D.S.M., Tápparo D.C., Rogovski P., Cadamuro R.D., de Souza E.B., da Silva R., Degenhardt R., Lindner J.D., Viancelli A., Michelon W. (2020). Hepatitis E Virus in Manure and Its Removal by Psychrophilic anaerobic Biodigestion in Intensive Production Farms, Santa Catarina, Brazil, 2018–2019. Microorganisms.

[72] Mulder A.C., Kroneman A., Franz E., Vennema H., Tulen A.D., Takkinen J., Hofhuis A., Adlhoch C., Members of HEVnet (2019). HEVnet: A One Health, collaborative, interdisciplinary network and sequence data repository for enhanced hepatitis E virus molecular typing, characterisation and epidemiological investigations. Eurosurveilance.

